# High Pathogenicity of Nipah Virus from *Pteropus lylei* Fruit Bats, Cambodia

**DOI:** 10.3201/eid2601.191284

**Published:** 2020-01

**Authors:** Maria Gaudino, Noémie Aurine, Claire Dumont, Julien Fouret, Marion Ferren, Cyrille Mathieu, Olivier Reynard, Viktor E. Volchkov, Catherine Legras-Lachuer, Marie-Claude Georges-Courbot, Branka Horvat

**Affiliations:** Centre International de Recherche en Infectiologie, CIRI, INSERM U1111, CNRS, UMR5308, Univ Lyon, University Claude Bernard Lyon 1, École Normale Supérieure de Lyon, Lyon, France (M. Gaudino, N. Aurine, C. Dumont, J. Fouret, M. Ferren, C. Mathieu, O. Reynard, V.E. Volchkov, M.-C. Georges-Courbot, B. Horvat);; ViroScan 3D, Trévoux, France (J. Fouret, C. Legras-Lachuer);; University Claude Bernard Lyon 1, LEM, UMR5557, CNRS, INRA, VetAgro Sup, Lyon (C. Legras-Lachuer);; Unité de Biologie des Infections Virales Emergentes, Institut Pasteur, INSERM P4, Jean Mérieux, Lyon (M.-C. Georges-Courbot)

**Keywords:** Nipah virus, henipavirus, emerging infection, *Pteropus* bats, fruit bats, spillover, phylogenetic analysis, sequencing, animal model, hamster, Cambodia, viruses, zoonoses, pathogenicity, NiV-Malaysia genotype, CSUR381, phosphoprotein, *Pteropus lylei*

## Abstract

We conducted an in-depth characterization of the Nipah virus (NiV) isolate previously obtained from a *Pteropus lylei* bat in Cambodia in 2003 (CSUR381). We performed full-genome sequencing and phylogenetic analyses and confirmed CSUR381 is part of the NiV-Malaysia genotype. In vitro studies revealed similar cell permissiveness and replication of CSUR381 (compared with 2 other NiV isolates) in both bat and human cell lines. Sequence alignments indicated conservation of the ephrin-B2 and ephrin-B3 receptor binding sites, the glycosylation site on the G attachment protein, as well as the editing site in phosphoprotein, suggesting production of nonstructural proteins V and W, known to counteract the host innate immunity. In the hamster animal model, CSUR381 induced lethal infections. Altogether, these data suggest that the Cambodia bat-derived NiV isolate has high pathogenic potential and, thus, provide insight for further studies and better risk assessment for future NiV outbreaks in Southeast Asia.

Nipah virus (NiV) is a zoonotic paramyxovirus that was first identified as the cause of an outbreak of encephalitis in humans in Malaysia and Singapore during 1998–1999 (*1*). Although NiV infection remains rare in humans, this virus has captured the attention of the public health community and scientists because of its high case-fatality rate, ranging from 40% in Malaysia to >90% in Bangladesh and India (*2*,*3*). Its high pathogenicity, potential for interspecies transmission, and lack of validated medical countermeasures led to the classification of NiV as a Biosafety Level 4 (BSL-4) pathogen. In 2015, the World Health Organization (WHO) listed NiV as a priority pathogen because of its probability of causing severe outbreaks and subsequently placed NiV on the WHO Blueprint list of priority diseases (*4*). This designation was strengthened by the NiV outbreak in Kerala, India, where the virus had not previously been reported (*3*).

NiV is a member of the *Henipavirus* genus, together with Hendra virus, which first emerged in Brisbane, Queensland, Australia, in 1994 (*5*), and the nonpathogenic Cedar virus, which was discovered in Australia in 2009 (*6*). In addition, full-length henipa-like viral sequences were found in fruit bats in Africa (*7*) and in rats in China (Moijang virus) (*8*). As part of the Mononegavirales, NiV has a nonsegmented, negative-sense, single-stranded RNA genome. *Pteropus* fruit bats, commonly known as flying foxes, are considered the henipavirus natural reservoir; these bats, when infected with NiV, do not seem to display any apparent clinical signs of disease (*9*).

Only 2 NiV lineages are known to circulate in Asia and cause disease in humans: NiV-Malaysia and NiV-Bangladesh. In India and Bangladesh, NiV transmission from bats to humans was shown to occur through the consumption of raw date palm juice or fruits contaminated with the saliva or urine from fruit bats (*10*). In addition, as observed during the outbreaks in Malaysia in 1998 and 1999, transmission can occur via contact with infected domestic animals, such as pigs, that act as amplifying hosts of the virus (*11*). Interhuman transmission has been reported in Bangladesh and India in >50% of NiV outbreaks (*12*).

Clinical manifestations of NiV infection in humans can range from asymptomatic to acute respiratory syndrome, generalized vasculitis, and fatal encephalitis. Among the few survivors of NiV outbreaks, long-term neurologic problems have been reported; 20% of patients have residual neurologic sequelae (*13*), and NiV-Malaysia–infected patients experienced relapse and late-onset encephalitis (*14*).

NiV and henipa-like viruses have been detected molecularly or serologically in *Pteropus* bats in different countries of Asia (*15*) and Africa (*7*), Australia (*16*), and Brazil (*17*), and the worldwide distribution of fruit bats poses a continuous threat to another spillover with possible pandemic potential (*18*). However, since 1998, all NiV cases in humans have been identified in Malaysia, India, Bangladesh, and the Philippines (*19*). Human cases of NiV have not been reported in Cambodia or neighboring countries since the first serologic detection of NiV in Cambodia and isolation of CSUR381 in *Pteropus lylei* bats in Cambodia in 2003 (*20*,*21*). Initial phylogenetic analyses of the nucleoprotein and attachment glycoprotein of CSUR381 suggested the virus was part of the NiV-Malaysia genotype (*21*). However, a full-genome characterization and phylogenetic analysis have not been performed. In addition, the growth dynamics and virulence of this virus have not been analyzed, thus limiting more comprehensive evaluation of this virus’s pathogenic potential. In this study, we performed an in-depth characterization of CSUR381, including its pathogenicity both in vitro and in vivo, ultimately to assess the outbreak risk that isolates circulating in Cambodia pose in Southeast Asia.

## Materials and Methods

### Viruses

In this study, we used 3 different NiV isolates: the NiV isolate CSUR381 from Cambodia (GenBank accession no. MK801755), NiV-Malaysia isolate UMMC1 (GenBank accession no. AY029767), and NiV-Bangladesh isolate SPB200401066 (GenBank accession no. AY988601). CSUR381 was isolated from *P. lylei* bat urine at the Pasteur Institut in Battambang, Cambodia, in 2003 (*21*), and the other 2 isolates were obtained from infected patients. We produced and titrated all viruses on Vero E6 cells.

### Full-Genome Sequencing

We amplified and titrated the Cambodia NiV isolate on Vero cells and, after the second cell passage, extracted viral RNA from supernatant using the QIAamp Viral RNA Mini Kit (QIAGEN, https://www.qiagen.com) according to the manufacturer’s instructions. We treated samples with DNase, purified and quantified RNA using the QuantiFluor RNA System (Promega, https://www.promega.com), and analyzed using the AATI High Sensitivity Genomic DNA Analysis Fragment Analyzer (Advanced Analytical Technologies Inc., https://www.agilent.com). Then, we amplified viral RNA using the Single Primer Isothermal Amplification Kit (NuGEN, https://www.nugen.com). We prepared a library using Ovation Ultra Low (NuGen), which gave us average DNA fragment sizes of 382–426 bp. We then sequenced the whole genome of CSUR381 using MiSeq Nano v2 (Illumina, https://www.illumina.com), which produced read lengths of 2 × 150 nt.

We carried out genome assembly de novo using SPAdes (http://cab.spbu.ru/software/spades) and predicted open reading frames with Prodigal (https://omictools.com/prodigal-tool). We reconstructed the missing 5′ and 3′ extremities using the 5′ RACE System for Rapid Amplification of cDNA Ends (Invitrogen, https://www.thermofisher.com) according to the manufacturer’s recommendations. We performed reverse transcription with SuperScript II Reverse Transcriptase (Invitrogen) using the primer GSP1-leader (5′-GACCATTGATCCAACATC-3′) to recover the viral leader sequence and GSP-trailer (5′-AAAGTGATTGTCTACTCACT-3′) to recover the trailer sequence. After column purification, we tailed cDNA sequences with cytidine triphosphate and terminal deoxynucleotidyl transferase. Last, we amplified dC-tailed cDNA using the Abridged Anchor Primer provided in the 5′ RACE System for Rapid Amplification of cDNA Ends Kit and primers nested-GSP2-leader (5′-TACAGCTTCAATGTCTGGGTCATT-3′) to amplify the viral leader sequence, and nested-GSP2-trailer (5′-CAAGTTCAAGGACACCAAAAGT-3′) to amplify the viral trailer sequence. We sequenced PCR products using Sanger technology and submitted the complete genome sequence of CSUR381 to GenBank (accession no. MK801755).

### Phylogenetic Analyses

Using the ClustalW algorithm (http://www.clustal.org), we performed multiple alignments for complete genomes and individual gene sequences. We implemented and manually checked the quality of alignments using BioEdit version 7.2.6 (*22*) and conducted genomic characterization and evolutionary analyses in MEGA version 7.0.26 (*23*). After determining the best DNA model to use for each alignment, we constructed maximum-likelihood phylogenetic trees for complete NiV genomes and all virus coding sequences. For statistical support, we used 500 bootstrap replicates for the analysis of the complete genome and 1,000 replicates for analyses of each gene.

### Cell Lines and Infection

We cultured NCI-H358 (human bronchioalveolar carcinoma) and Vero E6 (African green monkey kidney) cells in Dulbecco’s modified Eagle medium (DMEM) with GlutaMAX (Thermo Fisher Scientific, https://www.thermofisher.com) supplemented with 1% penicillin-streptomycin (10,000 U/mL), 1% L-glutamine, and 10% heat-inactivated (56°C for 30 minutes) fetal calf serum (FCS). We cultured human pulmonary microvascular endothelial cells (HPMECs) (*24*) in endothelial cell growth medium (Growth Medium MV 2 Kit; PromoCell, https://www.promocell.com). We incubated all these cell lines at 37°C with 5% carbon dioxide; all cell lines, including the *Pteropus* cell line described in the next paragraph, tested negative for *Mycoplasma* spp. by the MycoAlert kit (Lonza, https://www.lonza.com).

We generated a *Pteropus* flying fox cell line using a skin biopsy from the wing membrane of a female *P. giganteus* (also known as *P. medius* and flying fox) bat (*25*) of the order Yinpterochiroptera. Biopsies were collected from bats by Tiergarten Schönbrunn (Vienna, Austria) staff during regular veterinary checkups following appropriate guidelines to minimize animal stress. The biopsies were washed with sterile phosphate-buffered saline and transferred into Freezing Medium Cryo-SFM (PromoCell), and sample vials were put on dry ice for shipment to Centre International de Recherche en Infectiologie in Lyon, France. To obtain primary cell cultures, we fractionated the biopsies into petri dishes, harvested the homogenates, and incubated them at 37°C with 5% carbon dioxide in DMEM/F-12 (Gibco, https://www.thermofisher.com) supplemented with 10% fetal calf serum FCS, 1% L-glutamine (200 mM), 1,000 U/mL of penicillin, 1,000 U/mL of streptomycin, and 2.50 µg/mL amphotericin B (Gibco). We subsequently immortalized primary cells using the lentiviral vector SV40 large T-antigen produced at Genetic Analysis and Vectorology Platform (AniRA, École Normale Supérieure de Lyon, Lyon). We evaluated different clones on the basis of their morphologic stability and transfectability using jetPRIME kit (Polyplus, https://www.polyplus-transfection.com). We confirmed immortalization of clones by detecting large T-antigen inserts by reverse transcription PCR (RT-PCR). We cultured the final *Pteropus* cell line, which we designated PATGV1.12, in DMEM GlutaMAX supplemented with 10% heat-inactivated FCS. We additionally confirmed that this cell line was derived from *P. giganteus* bats by sequencing the mitochondrial region D-loop (*26*) and nuclear introns ACOX2, COPS7A, BGN, ROGD1, and STAT5A, which has been suggested to be pertinent for distinguishing among closely related bat species (*27*).

We infected cells in 12-well plates at 80% confluence with a multiplicity of infection (MOI) of 0.3. For virus replication kinetics studies, we took 4 time points postinfection into consideration: 0 h, 24 h, 48 h, and 72 h. We performed infections in BLS-4 facility Jean Mérieux (Lyon). For each time point, we collected cell lysates according to validated BSL-4 procedures. We collected supernatants and kept them at −80°C until titration by plaque assay on Vero E6 cells.

### Pseudotyping of Vesicular Stomatitis Virus and Evaluation of Cell Permissiveness

We used rVSVΔG-RFP (a recombinant vesicular stomatitis virus [VSV] in which the envelope glycoprotein G gene is replaced with the red fluorescent protein gene) (*28*,*29*) to generate pseudotyped VSVs harboring different combinations of NiV envelope glycoprotein G (attachment protein) and F (fusion protein) on their surfaces. Complementing rVSVΔG-RFP–infected cells with NiV glycoproteins expressed in trans, we were able to produce stocks of pseudotyped VSVs identical in their genetic background and differing only in the nature of their surface glycoproteins. Because the infectivity of rVSVΔG-RFP pseudotypes is restricted to a single round of replication, this tool is largely used for studying viral entry for a broad range of highly pathogenic viruses (*30*).

To create the pseudotypes, we cloned the NiV glycoprotein G and F genes from RNA isolated from CSUR381, UMMC1, and SPB200401066 into 6 separate pCAGGS plasmid vectors. We transfected these 3 plasmid pairs separately into BSR-T7 cells using TransIT-LT1 Transfection Reagent (Mirus Bio, https://www.mirusbio.com). We infected cells with rVSVΔG-RFP 16 h after transfection to produce a pseudotyped VSV for each NiV isolate. We collected supernatants at 24 h postinfection and concentrated pseudotyped VSVs by ultracentrifugation (28,000 rpm for 2 h at 4°C). We titrated these viruses on Vero cells. To evaluate viral entry into different cell lines, we performed infections in 24-well plates using 80% confluent, adherent cells and a 1-h contact between virus and cells. We determined the percentage of cells infected 6 h postinfection by quantifying cells expressing RFP via flow cytometry on a BD LSRFortessa (https://www.bd.com).

### RNA Extraction and Real-Time RT-PCR

At the indicated time points, we collected cells and extracted RNA using the NucleoSpin RNA Kit (Macherey-Nagel, https://www.mn-net.com) according to the manufacturer’s instructions. We assessed the yield and purity of extracted RNA using the DS-11-FX spectrophotometer (DeNovix, https://www.denovix.com). We reverse transcribed extracted RNA using the iScript Select cDNA Synthesis Kit (Bio-Rad, https://www.bio-rad.com) and performed real-time PCR using Platinum SYBR Green qPCR SuperMix-UDG (Invitrogen) on a StepOnePlus Real-Time PCR System (Applied Biosystems, https://www.thermofisher.com). As previously described (*31*), we amplified the NiV nucleoprotein gene and the *Pteropus* glyceraldehyde 3-phosphate dehydrogenase housekeeping gene using forward primer 5′-ATCATCCCTGCTTCTACT-3′ and reverse primer 3′AGGTCAGATCCACAACT-5′. We analyzed quantitative RT-PCR results using StepOne version 2.3 (Applied Biosystems).

### Experimental Infection of Hamsters

We obtained 2-month-old male golden hamsters (*Mesocricetus auratus*) from Janvier Labs (https://www.janvier-labs.com). We housed hamsters in a BSL-4 containment facility (INSERM P4, Jean Mérieux, Lyon) and handled them according to the regulations for animal maintenance of France. We treated hamsters with isoflurane anesthesia before manipulations. We subcutaneously infected 2 groups of 6 hamsters with a high dose (13,500 PFU/animal) of either the NiV-Malaysia or Cambodia NiV isolate and followed hamsters daily to record their body temperature and weight. The regional ethics committee for animal experimentation (Lyon) approved these animal experiments.

## Results

### Full-Genome Characterization and Phylogenetic Analyses

Analysis of the assembled viral sequence of CSUR381 showed a total genome length of 18,246 nt, similar to the lengths of NiV isolates reported in Malaysia. The nucleotide composition was 27.8% T or U, 18.4% C, 33.6% A, and 20.2% G; total GC content was 38.6%. To investigate genetic relationships between CSUR381 and other henipaviruses, we constructed distance matrices for the complete genome and for each gene using the p-distance method. When we compared the sequence of CSUR381 with those of other NiVs available in GenBank, the most similar sequences (with 97.7% nucleotide identity) were from NiV-Malaysia human isolates (GenBank accession nos. NC002728.1 and AY029768.1; Table 1). We also calculated nucleotide identity and amino acid homology for each of the 6 structural genes (Table 2). Genetic pairwise comparisons with other NiV isolates showed the lowest nucleotide identity and amino acid homology for phosphoprotein (087.1/82.7%) and highest for matrix protein (98.9/99.4%).

**Table 1:** Whole-genome pairwise nucleotide identity comparisons between Nipah virus CSUR381, Cambodia, 2003, and other available henipaviruses

Henipavirus (GenBank accession no.)	Nucleotide identity, %*
Nipah/Malaysia/2000/human (NC002728.1)	97.7
Nipah/Malaysia/2001/human (AY029768.1)	97.7
Nipah/Malaysia/1999/swine (AJ627196.1)	97.6
Nipah/Bangladesh/2004/human (AY988601.1)	91.6
Nipah/India/2007/human (FJ513078.1)	91.4
Nipah/Bangladesh/2008/human (JN808863.1)	91.4
Nipah/India/2018/human (MH396625.1)	91.3
Hendra/Australia/2008/human (JN255805.1)	70.0
Hendra/Australia/2009/bat (JN255803.1)	69.9
Hendra/Australia/2007/horse (HM044321.1)	69.9
Cedar/Australia/2009/Ptalecto (JQ001776.1)	56.7
Paramyxo/Ghana/2009/Eidolonhelvum (HQ660129.1)	52.9
Mojiang/China/2012/Rattusflavipectus (NC025352.1)	48.9

*Calculated by using the p-distance method.

**Table 2:** Pairwise comparison of NiV CSUR381, Cambodia, 2003, and other available NiVs, by NiV gene*

NiV (GenBank accession no.)	NiV gene, % nucleotide identity/% homology of deduced amino acid†					
	N	P	M	F	G	L
Nipah/Malaysia/2010/Pvampyrus (FN869553.1)	98.3/99.2	96.3/94.9	98.9/99.4	98.4/98.7	97.1/98.5	98.0/99.3
Nipah/Malaysia/2000/human (NC002728.1)	97.9/98.7	95.9/94.1	98.5/99.1	98.1/98.9	97.1/98.5	98.1/99.4
Nipah/Bangladesh/2004/human (AY988601.1)	93.8/98.5	87.8/84.8	93.0/99.1	93.0/98.2	88.3/95.5	91.7/98.2
Nipah/Bangladesh/2008/human (JN808863.1)	93.9/98.7	87.6/84.4	93.0/99.1	93.3/98.5	88.1/95.5	91.8/98.4
Nipah/India/2007/human (FJ513078.1)	93.5/98.5	87.4/84.3	92.9/98.9	93.1/98.4	88.0/95.3	91.9/98.4
Nipah/India/2018/human (MH396625.1)	93.3/98.5	87.1/82.7	92.6/99.1	93.0/98.5	87.2/95.3	91.7/98.5
Nipah/Thailand/2010/Plylei (KT163252.1)	93.7/98.7					
Nipah/Thailand/2010/Phypomelaneus (KT163247.1)	97.8/99.0					

*F, fusion protein; G, attachment protein; L, polymerase; M, matrix protein; N, nucleoprotein; NiV, Nipah virus; P, phosphoprotein. †Both calculated by using the p-distance method.

**Figure 1:**
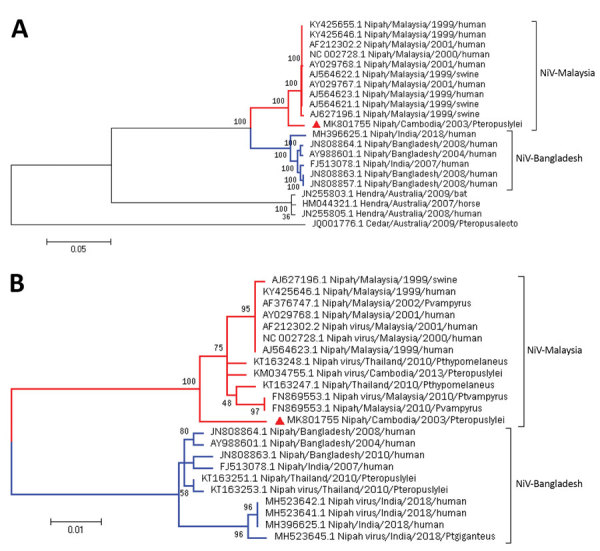
Maximum-likelihood phylogenetic analysis of NiV CSUR381, Cambodia, 2003 (red triangle), compared with other henipaviruses and NiVs. A) Phylogenetic tree constructed with complete genome sequences. A general time-reversible model was calculated as the best DNA model to conduct this analysis. B) Phylogenetic tree constructed by using the nucleocapsid gene. The Kimura 2-parameter model was calculated as the best DNA model to conduct this analysis. Bootstrap statistical support is marked on branch nodes. GenBank accession numbers of isolates are provided in branches, and NiV lineages of isolates are indicated. Phylogenetic trees are drawn to scale; scale bars represent branch lengths measured in the number of substitutions per site. NiV, Nipah virus.

Using the maximum-likelihood method, we constructed phylogenetic trees on the basis of the complete genome (Figure 1, panel A) and the nucleocapsid gene (Figure 1, panel B). The general time-reversible model for the complete genome and the Kimura 2-parameter model for the nucleocapsid gene were predicted to be the best for performing those particular phylogenetic analyses. CSUR381 clustered with the monophyletic group of the NiV-Malaysia genotype for both the whole genome and nucleocapsid gene; bootstrap support was >98% in all cases, confirming the previous partial genomic characterization of CSUR381 (*24*). We then generated phylogenetic trees for each of the coding sequences of the 6 NiV structural proteins, which gave equivalent results (Appendix Figure 1).

**Figure 2:**
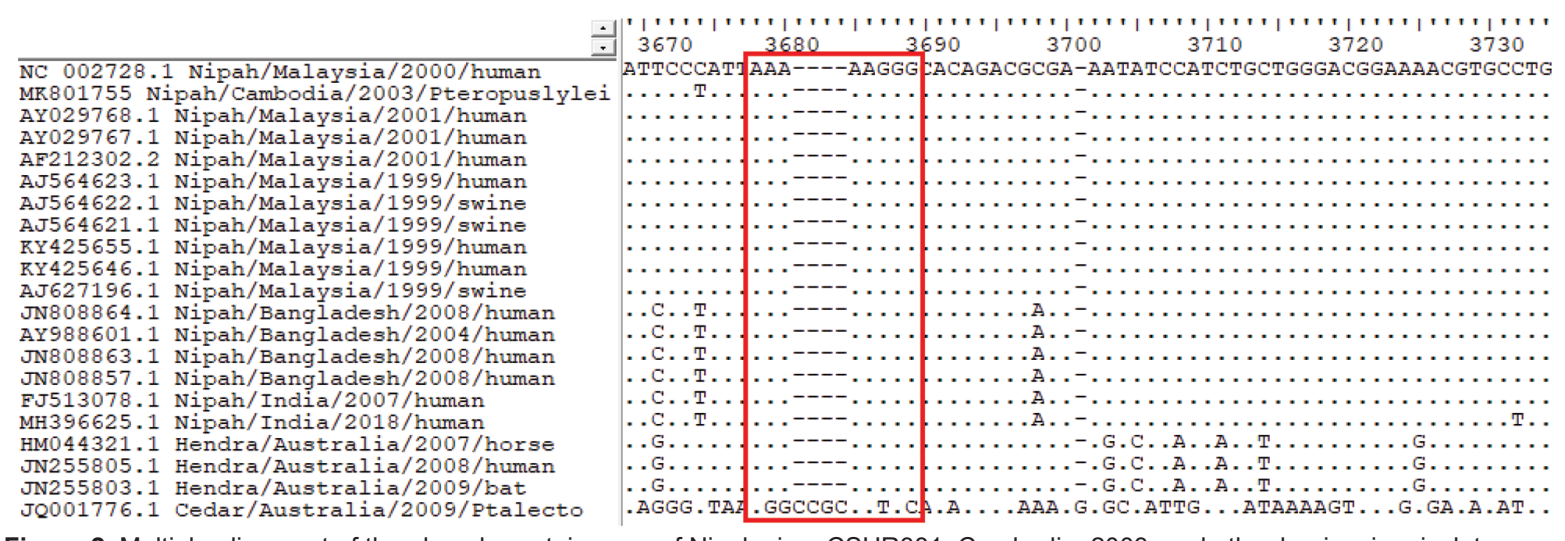
Multiple alignment of the phosphoprotein gene of Nipah virus CSUR381, Cambodia, 2003, and other henipavirus isolates. The highly conserved editing site (5′-AAAAAGGG-3′, red outline) is present in all Nipah and Hendra virus sequences but absent in the nonpathogenic Cedar virus sequence. GenBank accession numbers are provided for all isolates.

Multiple alignment of the henipavirus phosphoprotein gene (Figure 2) revealed high conservation of the editing site (5′-AAAAAGGG-3′) in CSUR381, similar to other NiV and Hendra virus isolates and different from Cedar virus, a nonpathogenic virus isolated from a *P. alecto* bat in Australia (*6*). This finding suggests that CSUR381 might produce the nonstructural proteins V and W, capable of interacting with the host innate cellular immune response (*32*). Comparisons of the deduced V, W, and C amino acid homologies between CSUR381 and other known NiVs showed a variation of 88%–100% (Appendix Table).

### Evaluation of Virus Entry

**Figure 3:**
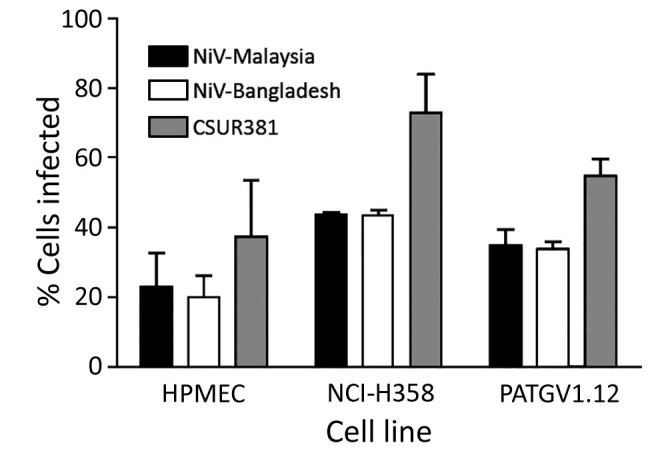
Evaluation of entry of VSVΔG-RFPs (vesicular stomatitis virus in which the envelope glycoprotein G gene is replaced with the red fluorescent protein gene) pseudotyped with the surface glycoproteins of NiVs CSUR381 (Cambodia 2003 isolate), UMMC1 (NiV-Malaysia isolate), and SPB200401066 (NiV-Bangladesh isolate) in different cell types. Infections of HPMEC, NCI-H358 (human bronchioalveolar cells), PATGV1.12 (bat cells), and Vero cells were performed at a multiplicity of infection of 0.3, and the percentages of infected cells were evaluated 6 hours postinfection by measuring RFP by flow cytometry and normalizing values to those from Vero cells. Histograms indicate the mean of 3 independent experiments, and error bars indicate upper half of SD. HPMEC, human pulmonary microvascular endothelial cell; NiV, Nipah virus.

We next determined the cellular permissiveness of a human endothelial cell line (HPMEC), a human respiratory epithelial cell line (NCI-H358), the newly generated *Pteropus* bat cell line (PATGV1.12), and Vero cells to CSUR381 compared with the NiV-Malaysia (UMMC1) and NiV-Bangladesh (SPB200401066) isolate using pseudotyped rVSVΔG-RFP viruses. Cell lines were infected for 1 h at an MOI of 0.3. The percentages of cells infected were analyzed by flow cytometry 6 h after infection (Figure 3), and results from HPMEC, NCI-H358, and PATGV1.12 were normalized to the findings from Vero. All tested cell lines were permissive to infection with all 3 viruses tested. Entry of NiV pseudotypes into the bat cell line PATGV1.12 and human respiratory epithelial cell line was similar. Compared with the NiV-Malaysia and NiV-Bangladesh pseudotypes, the CSUR381 pseudotyped virus showed higher but not significantly increased entry into the 3 tested cell lines (1-way analysis of variance).

We further analyzed the amino acid sequences of the F and G proteins of the 3 viruses by multiple alignment. The glycosylation site (N529/Q530/T531) (*33*) and ephrin-B2 and ephrin-B3 binding sites (*34*) in the G attachment protein were preserved (Appendix Figure 2). In addition, multiple alignments showed that the F cleavage site was preserved among all analyzed NiV isolates (Appendix Figure 3). Last, an analysis of the predicted N-terminal and C-terminal heptad-repeat regions within the F protein, which are needed for NiV fusion (*35*), showed high conservation, and compared with NiV-Malaysia and NiV-Bangladesh, only 1 aa difference (V159→I) was detected in CSUR381 (Appendix Figure 3). Altogether, the high conservation of the NiV glycoproteins and results from pseudotype virus studies suggest that CSUR381 can enter target cells at least as well as NiV-Malaysia and NiV-Bangladesh.

### Replication of NiV Isolates in Different Cell Types

**Figure 4:**
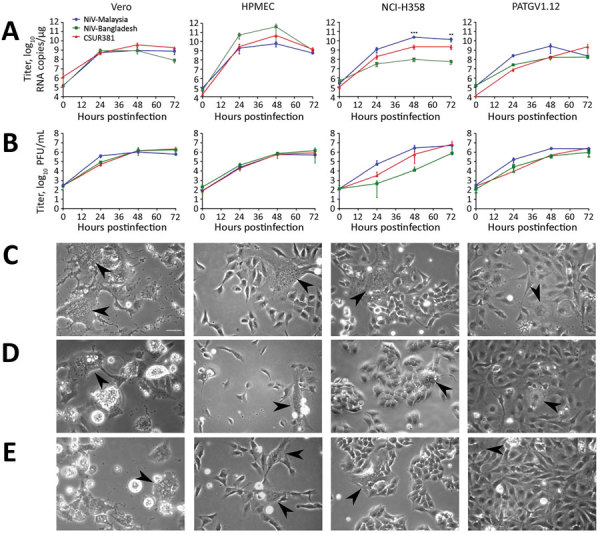
Replication of NiVs CSUR381 (Cambodia 2003 isolate), UMMC1 (NiV-Malaysia isolate), and SPB200401066 (NiV-Bangladesh isolate) in Vero, HPMEC, NCI-H358 (human bronchioalveolar cells), and PATGV1.12 (bat cells). A) Real-time reverse transcription PCR titer. Cells were infected at a multiplicity of infection of 0.3, and the production of the nucleocapsid gene was measured. Significance was measured by 2-way analysis of variance. B) Kinetics of infectious virus particle production in supernatant measured by Vero plaque assay. The average of 2 independent experiments is presented. C–E) Cytopathic effect of observed by light microscopy 48 h after infection with the NiV-Malaysia isolate (C), the NiV-Bangladesh isolate (D), and CSUR381 (E). Giant multinucleated cells are indicated with arrowheads. Scale bar indicates 25 µm. HPMEC, human pulmonary microvascular endothelial cell; NiV, Nipah virus. **p<0.01; ***p<0.001.

To further evaluate the virulence of CSUR381, we compared the replication kinetics of this virus with those of the NiV-Malaysia and NiV-Bangladesh isolates. We infected cell types known to be primary targets of NiV in humans, pulmonary endothelial (HPMEC) and bronchioalveolar epithelial (NCI-H358) cells, and the bat cell line PATGV1.12 at an MOI of 0.3 (Figure 4). NiV RNA synthesis was highest in HPMEC, where NiV-Bangladesh replicated the best, although a similar level of RNA and infectious virus particle production was observed for all 3 viruses (Figure 4, panel A). In accordance with virus entry studies (Figure 3), virus replication was also observed in PATGV1.12 (Figure 4, panels A and B). Differences among the 3 tested NiV isolates were observed only in NCI-H358, where NiV-Malaysia RNA synthesis was significantly increased (p<0.001 by 2-way analysis of variance) compared with NiV-Bangladesh, provoking remarkable cytopathic effects (Figure 4, panel C). The formation of giant multinucleated cells, a hallmark of NiV infection, were already visible at 24 hours postinfection in all cell types and further developed during the course of the infection (Figure 4, panels C–E). Vero cells showed the most visible cytopathic effects, probably because of their interferon incompetence (*36*).

### Experimental Infection of Hamsters

We compared the pathogenicities of CSUR381 and the NiV-Malaysia isolate using the golden hamster animal model (*37*). We infected 6 hamsters with either CSUR381 or the NiV-Malaysia isolate and followed them for clinical signs of infection. At 6 days postinfection, the first neurologic signs (which included paralysis and trembling limbs) were observed in both groups; their presentation rapidly evolved toward breathing difficulties and prostration. Weight reductions were evident in several animals in the late stages of infection (Figure 5, panel A), and decreases in body temperature were found in a few hamsters (Figure 5, panel B). At 7 days postinfection, 100% lethality was observed in the CSUR381 group. In the Malaysia group, 1 animal survived until 10 days postinfection (Figure 5, panel C); however, the difference between the 2 groups was not significant. These results demonstrate similar lethality of the 2 analyzed NiV isolates, supporting our other data and suggesting CSUR381 has a high pathogenic potential.

## Discussion

In this study, we performed a molecular and genetic characterization of CSUR381, a NiV isolated from *P. lylei* bats in Cambodia. Furthermore, we analyzed its pathogenicity compared with those of 2 other NiV isolates derived from human patients from the Malaysia and Bangladesh outbreaks. Our results highly suggest that CSUR381 is part of the NiV-Malaysia genotype. Further phylogenetic comparisons with other NiV isolates demonstrated 83%–99% amino acid homology for each of the 6 structural proteins. In addition, the editing site of the phosphoprotein gene was preserved, suggesting possible production of the nonstructural V and W proteins known to be involved in counteracting the host innate immune system and thus contributing to pathogenicity of CSUR381 (*32*).

Our virus entry studies showed highly similar results among the NiVs tested. All isolates entered *Pteropus* bat and human cell lines at similar levels; high conservation of the NiV entry receptors (ephrin-B2 and ephrin-B3) (*38*) might be responsible for the observed results. Our data also indicate that CSUR381 enters all tested cell types as well as the other 2 NiV isolates tested, suggesting that virus entry is not a limiting factor preventing CSUR381 spillover from bats to humans. In addition, all 3 tested NiV isolates infected cells and replicated in bat and human cell lines at similar levels. Results of infections with CSUR381 in hamsters additionally strengthened the notion that CSUR381 is possibly similar pathogenically to the tested NiV-Malaysia strain, which caused fatal outbreaks in Malaysia (*1*).

Although NiV has been shown to circulate in Cambodia (*20*,*21*), Thailand (*39*), and Vietnam (*40*), transmission to humans or domestic animals has not been reported in these countries. According to our results, the absence of detected outbreaks in this region cannot be attributed to lower pathogenicity of the circulating NiVs; our results suggest that other factors probably contribute. However, the NiV isolate presented in this report has been the only live NiV isolated in this region, and the existence of other NiVs with different pathogenic potentials cannot be excluded.

In Cambodia, *P. lylei* bats were found to often forage in residential areas and visit palm trees used in the region as a source of date palm sap; thus, opportunities abound for bats to interact with humans and livestock in this country (*41*). Bat colony migration toward urban sites is further enhanced by the presence of hunters in rural areas (*42*) and deforestation (causing consequent damage to roosting trees and food sources) (*43*). Contamination of palm sap, which is consumed raw by persons in the region, with bat urine, saliva, or feces was found to be a major route of NiV transmission to humans during annual outbreaks in Bangladesh (*10*).

Diverse agricultural practices in Southeast Asia could also play a role in NiV regional ecodynamics, potentially favoring easier NiV spillover in some countries over others. High-intensity pig farming was recognized as a major risk factor for outbreaks in Malaysia during 1998–1999; because of the low-scale pig production ongoing in Cambodia (*44*), the risk for NiV transmission from *Pteropus* spp. to domestic animals and humans in this country might be reduced.

Unrecognized NiV outbreaks might have occurred in Cambodia and neighboring countries; hospital-based surveillance in Bangladesh was shown to have missed nearly half of the NiV outbreaks in that country since the first reported virus emergence (*45*). Interdisciplinary approaches are certainly required to identify these outbreaks and the drivers of NiV emergence (*46*), and regular testing of patients with encephalitis in Cambodia and neighboring countries could provide additional insight. Our study contributes to the assessment of the risk for NiV outbreaks in Asia. Our findings can be used to help target adequate preventive measures, which could ultimately help reduce the risk for NiV emergence.

Appendix More information about high pathogenicity of Nipah virus from *Pteropus lylei* fruit bats, Cambodia.
