# Supplementary material for: High Pathogenicity of Nipah Virus from Pteropus lylei Fruit Bats, Cambodia
Source: Emerg Infect Dis. 2020 Jan;26(1):104–13. doi: 10.3201/eid2601.191284 (PMC6924896; doi:10.3201/eid2601.191284)
Supplement: Appendix — More information about high pathogenicity of Nipah virus from Pteropus lylei fruit bats, Cambodia. [file 19-1284-Techapp-s1.pdf]

Article DOI: https://doi.org/10.3201/eid2601.191284

High Pathogenicity of Nipah Virus from *Pteropus lylei* Fruit Bats, Cambodia

Appendix

Appendix Table. Genetic pairwise comparisons of NiV nonstructural proteins V, W, and C between NiV/Cambodia and other NiV isolates*

NiV, GenBank accession no.	Nonstructural NiV proteins, % nucleotide identity/% homology of deduced amino acid		
	V	W	C
Nipah/Malaysia/2000/human, NC_002728.1	97.2/95.7	97.3/95.8	98.6/98.2
Nipah/Malaysia/2010/Pvampyrus, FN869553.1	97.1/95.5	96.6/94.0	99.4/100
Nipah/Bangladesh/2004/human, AY988601.1	92.4/89.4	92.5/89.6	97.6/97.0
Nipah/Bangladesh/2008/human, JN808863.1	91.8/88.5	91.9/88.7	97.4/97.0
Nipah/India/2007/human, FJ513078.1	92.1/88.9	92.2/89.1	97.6/97.0
Nipah/India/2018/human, MH396625.1	91.7/88.0	91.8/88.0	97.2/96.4

* NiV, Nipah virus.
†The nucleotide identity and deduced amino acid homology were calculated using p-distance method.

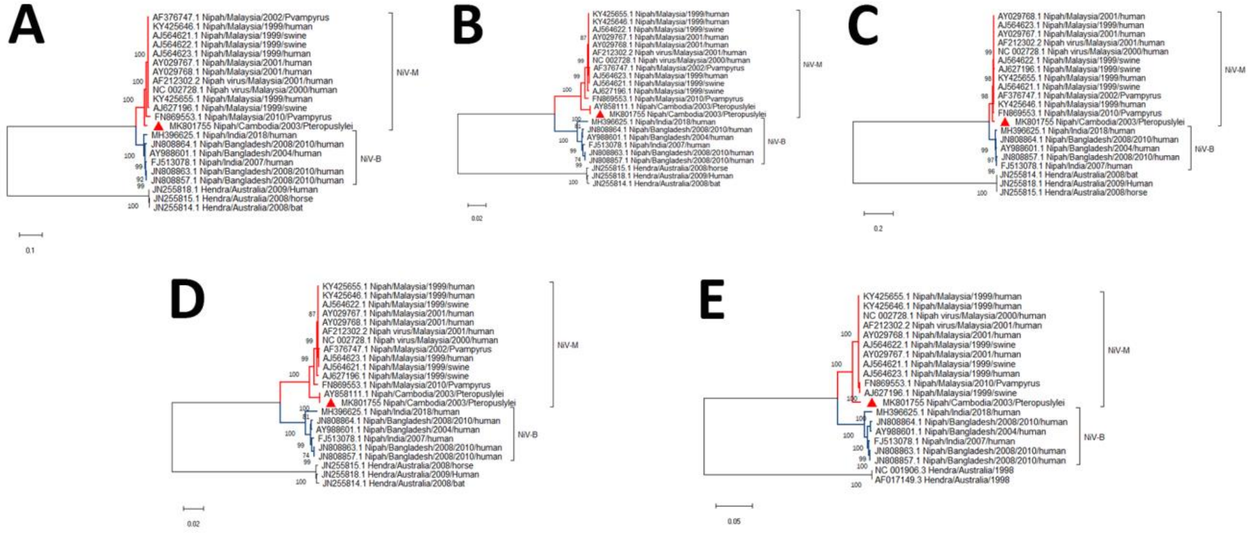

Appendix Figure 1. Maximum-likelihood phylogenetic trees based on genes coding for *Henipavirus.* A) Phosphoprotein, B) Matrix protein, C) Fusion protein, D) Attachment glycoprotein, and E) Polymerase. The Cambodia 2003 Nipah virus isolate CSUR381 (GenBank MK801755, red triangle) clusters with NiV-Malaysia isolates. Bootstrap statistical support is shown on branch nodes. Hasegawa-Kishino-Yano model and Tamura-3-parameter were calculated as the best DNA model to conduct the analysis for these genes. The phylogenetic trees are drawn to scale and scale bar represents branch lengths measured in the number of substitutions per site.

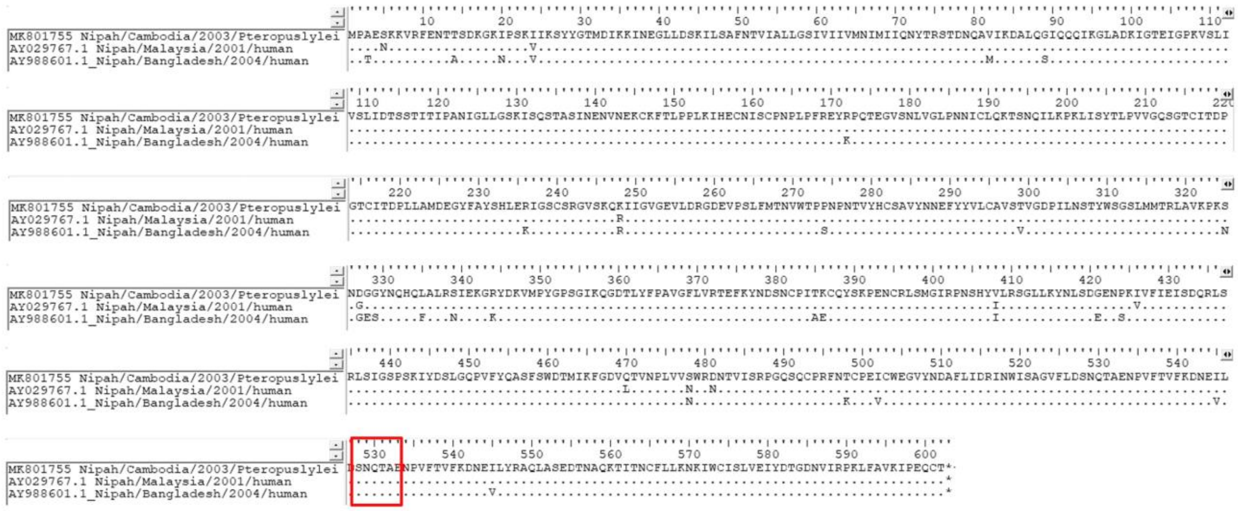

Appendix Figure 2. Amino acid multiple alignment of the attachment glycoprotein (G) of three Nipah virus (NiV) isolates used in the study. Specific amino acid changes were found in NiV/Cambodia G sequence (V24→I, R248→K, G327→D, I408→V, N478→S). The glycosylation site in G attachment protein (N529/Q530/T531) is conserved among the three tested isolates (marked in red). Polymorphism at Ephrin B2 and B3 potential binding sites was not observed (W504, Q530, T531, A532, and N557, E505, E533, respectively).

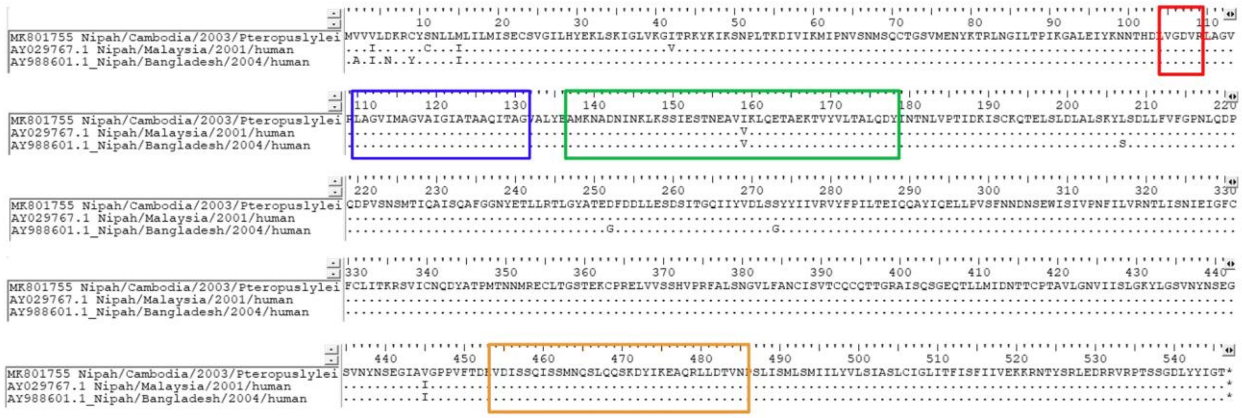

Appendix Figure 3. Amino acid multiple alignment of the fusion protein (F) of three Nipah virus (NiV) isolates used in the study. Specific NiV/C amino acid changes were found in the beginning of F2 subunit (I4→V, I15→M). Fusion protein cleavage region (N104-R109, marked in red) and fusion peptide (L110-V133, marked in blue) are preserved among all analyzed NiV isolates. Predicted N-terminal and C-

terminal heptad repeat regions (HRN and HRC) are marked in green and orange respectively. V159→I mutation was observed in HRN region, while no variability was found within the HRC.
